# Racial differences in patterns of treatment among men diagnosed with de novo advanced prostate cancer: A SEER‐Medicare investigation

**DOI:** 10.1002/cam4.2092

**Published:** 2019-05-15

**Authors:** Jennifer L. Beebe‐Dimmer, Julie J. Ruterbusch, Kathleen A. Cooney, Adam Bolton, Kendra Schwartz, Ann G. Schwartz, Elisabeth Heath

**Affiliations:** ^1^ Barbara Ann Karmanos Cancer Institute Detroit Michigan; ^2^ Department of Oncology Wayne State University School of Medicine Detroit Michigan; ^3^ Duke University School of Medicine and Duke Cancer Institute Durham North Carolina

**Keywords:** African American, AJCC Stage IV, prostate cancer, treatment disparities

## Abstract

**Purpose:**

Approximately 5% of men were initially diagnosed with (also referred to as de novo) advanced stage prostate cancer and experience far poorer survival compared to men diagnosed with local or regionally advanced disease. Given the number of new therapies targeting metastatic and castrate‐resistant disease, we sought to describe recent treatment patterns by race for de novo AJCC stage IV prostate cancer.

**Methods:**

We used Surveillance, Epidemiology, and End Results (SEER) data linked to Medicare files to identify men aged 66 and older diagnosed in 2004‐2014 with advanced prostate cancer, and examined patterns of treatment among all patients and stratified by race/ethnicity.

**Results:**

There were 8828 eligible patients identified, and non‐Hispanic black (NHB) patients were more likely to go without treatment (*P* < 0.001) compared to non‐Hispanic white (NHW) patients, even after accounting for early mortality and TNM stage. The frequency of nearly all forms of treatment was lower among NHB with the exception of orchiectomy, which was significantly higher (10.1% vs 6.1%, *P* < 0.001), and the use of the progesterone Megace among Medicare Part D enrollees (24.6% vs 15.0%, *P* < 0.001).

**Conclusions:**

Results from this study of elderly Medicare patients presenting with advanced stage prostate cancer suggest that NHB men are less likely to pursue aggressive treatment options. With the reduction in screening for prostate cancer, presumably tied to USPSTF recommendations, and the increasing incidence of men diagnosed with de novo metastatic disease, understanding drivers of treatment‐related decisions are critical in reducing racial disparities in advanced prostate cancer outcomes.

## 1 INTRODUCTION

Prostate cancer is the most common cancer diagnosed among men in the United States with the vast majority of men diagnosed with organ‐confined disease. For these men, both 5‐ and 10‐year relative survival rates approach 100%. Unfortunately, approximately 5% of men are initially diagnosed with metastatic disease and the 5‐year relative survival rate for this group drops to just 29%.^1^ Recent data indicate that there has been a significant rise in the incidence of metastatic prostate cancer particularly in younger men.^2^, ^3^

Non‐Hispanic black (NHB) men are 70% more likely to be diagnosed with prostate cancer and are 2.4 times more likely to die from their disease compared to non‐Hispanic whites (NHW).^4^ Just over 30% of all new prostate cancer cases and 12% of deaths occur in NHB men.^4^ We no longer observe differences by race in either tumor stage at the time of diagnosis or 5‐year relative survival by stage at diagnosis.^4^ Therefore, the excess mortality experienced by NHB must occur farther out from the time of diagnosis and is likely attributed to cause other than prostate cancer.^1^, ^4^, ^5^ Fortunately, mortality for NHB men with prostate cancer has declined significantly over the past 25 years at a rate that is approximately parallel to that observed in NHW men.^4^, ^6^ The racial disparity in mortality has been attributed in part to differences in access to medical care and quality of treatment.

Historically, for men who were either initially diagnosed with or develop metastatic prostate cancer, androgen deprivation therapy (ADT) is the most widely used treatment to slow the progression of disease.^7^ However, if these men survive long enough, all prostate cancer eventually becomes castrate resistant. When this happens, both treatment options and their effectiveness become much more limited. Interestingly, there is evidence to suggest that local treatment of metastatic prostate cancer, such as surgery or radiation therapy, improves overall survival for patients.^8^, ^9^ Moreover, there have been a number of new therapies approved in the past 5‐6 years based upon very promising clinical trial data,^10^, ^11^ but we know little about the prevalence and correlates of use of these newer treatments in the general population.

Therefore, the goal of the current investigation is to describe racial differences in the pattern and prevalence of elected treatments among men first diagnosed with advanced stage prostate cancer and correlates of treatment. To accomplish this, we analyzed the most recent data available from nearly 9000 elderly men diagnosed with de novo AJCC Stage IV prostate cancer from the SEER program registry with linkage to Medicare claims files.

## 2 METHODS

The SEER‐Medicare dataset links two population‐based sources of data to provide information about the experience of elderly cancer patients in the United States (US). The SEER program, sponsored by the National Cancer Institute, is a network of population‐based cancer registries that routinely collects information on patients diagnosed with invasive cancer residing within one of the registry catchment areas. SEER is composed of 18 statewide or regional cancer registries, collecting data on patient demographics, tumor histology and pathology, first course of treatment, and survival. By linking SEER registry data to Medicare enrollment and claims information, the SEER‐Medicare database provides additional information on treatment and outcomes of approximately 25% of all elderly patients diagnosed with cancer in the US

**Figure 1:**
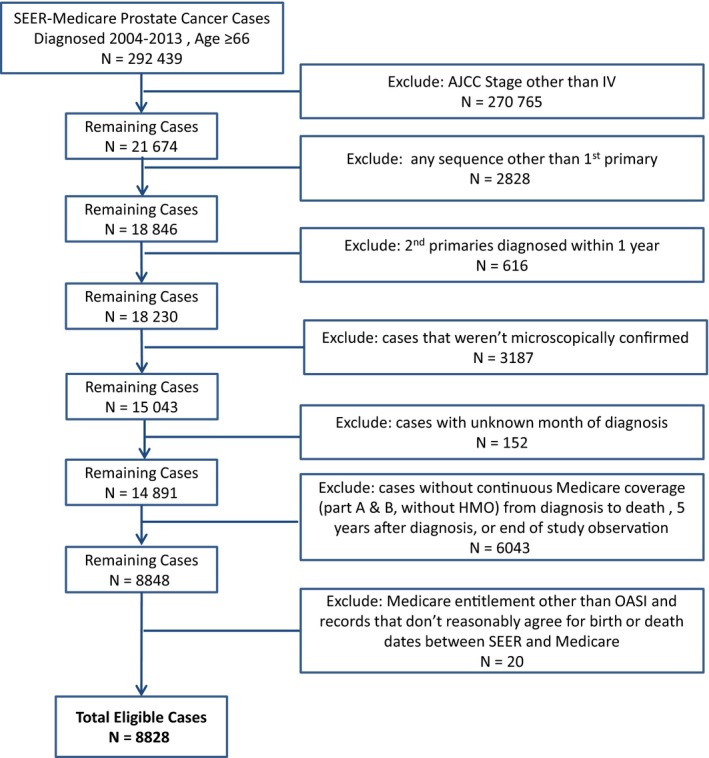
Study inclusion and exclusion criteria for identifying men diagnosed at denovo AJCC stage IV prostate cancer from SEER‐Medicare files

Patient eligibility is summarized in Figure 1 and includes those diagnosed between 2004 and 2014 with first primary AJCC stage IV (6th Edition) prostate cancer and living in one of 16 SEER catchment areas (Atlanta, Connecticut, Hawaii, Iowa, New Mexico, Utah, Rural Georgia, Detroit, Seattle‐Puget Sounds, Los Angeles, San Jose‐Monterey, San Francisco‐Oakland, Greater California, Kentucky, Louisiana, and New Jersey). Patients in the special population registries (Alaska Native and Arizona Indians) are not included in SEER‐Medicare. Patients aged 66 and older were selected to document the presence of any comorbid conditions (summarized using a modified Charlson Comorbidity Index) in the 12 months prior to diagnosis. Patients not continuously enrolled in both Part A and B Medicare, or enrolled in a health maintenance organization (HMO), from 12 months prior to diagnosis until death or 31 December 2014, were excluded to minimize the potential for missing information due to claims not processed through Medicare. Additional exclusion criteria include: (a) diagnosis of a second primary cancer within 1 year of prostate cancer diagnosis, (b) diagnosis not microscopically confirmed, (c) eligibility for Medicare not age‐related, (d) birth or death date discrepancies between SEER and Medicare, and (e) missing diagnosis month. Based upon these criteria, 8828 patients were eligible for the study.

All treatments within the first month of diagnosis until death or end of study observation were identified using Medicare claims data (relevant HCPCS and ICD‐9 codes are listed in Supplemental Table S1). Among all men, receipt of a radical prostatectomy, transurethral resection of the prostate (TURP), radiation therapy, chemotherapy, ADT (both chemical and surgical), zoledronic acid, Sipuleucel‐T, denosumab, radium‐223, and cryotherapy were identified. Men who did not receive any of the treatments above were considered untreated (excluding oral medications). Among the subset of men who had continuous Part D benefits (prescription drug coverage) during the study observation period, the receipt of the following oral medications were identified: bicalutamide, enzalutamide, abiraterone acetate, megestrol acetate, dutasteride, and finasteride.

### 2.1 Statistical methods

Statistical analyses were performed using Statistical Analysis Software (SAS Institute v.9.4, Cary, NC) and R package “stddiff,”^12^ and a *P*‐value < 0.05 was considered statistically significant. The distribution of demographic and clinical variables was summarized for all patients and stratified by race/ethnicity. The distribution of variables was compared using chi‐square tests for categorical variables and Cochran‐Armitage test for ordinal variables, and the standardized difference was calculated to summarize the magnitude of the differences. The proportion of men who received each treatment type was compared by race/ethnicity using chi‐square tests for all men and among the subset of men who had Part D benefits. Sensitivity analyses were conducted (a) excluding men who died within 3 months of diagnosis and (b) including only men diagnosed with confirmed metastatic (M1) disease. The third analysis stratified the results by age at diagnosis (age 66‐79 vs age 80 or older). Logistic regression was used to estimate the odds and 95% confidence intervals for not receiving prostate cancer treatment (excluding oral medications) for each of the demographic and clinical variables listed in Table 1. A multivariate logistic regression model for not receiving treatment was based on a stepwise regression with an inclusion and exclusion alpha set at 0.05.

**Table 1:** Demographic and clinical characteristics for prostate cancer cases diagnosed at advanced stage in the SEER‐Medicare database by race and ethnicity

	All patients		NHW		NHB		*P* value	Standardized Difference	Other	
	N	%	N	%	N	%			N	%
Total	8828		6617		1131				1080	
Age group							<0.001	0.29		
66‐69	1932	22	1359	21	321	28			252	23
70‐74	2135	24	1532	23	296	26			307	28
75‐79	1779	20	1314	20	235	21			230	21
80‐84	1529	17	1212	18	159	14			158	15
85+	1453	16	1200	18	120	11			133	12
Marital status^a^							<0.001	0.52		
Single (never married)	936	11	584	9	246	22			106	10
Married (or equivalent)	5444	62	4240	64	484	43			720	67
Separated or Divorced	694	8	461	7	152	13			81	8
Widowed	1208	14	934	14	167	15			107	10
Year of Diagnosis							0.086	0.06		
2004‐2006	2745	31	2055	31	370	33			320	30
2007‐2009	2453	28	1800	27	323	29			330	31
2010‐2013	3630	41	2762	42	438	39			430	40
Poverty indicator^a^(based on census tract)							<0.001	1.13		
0%‐<5% poverty	1908	22	1667	25	65	6			176	16
5% to <10% poverty	2173	25	1868	28	118	10			187	17
10% to <20% poverty	2299	26	1699	26	287	25			313	29
20% to 100% poverty	1717	19	853	13	606	54			258	24
Histology							0.026	0.08		
Adenocarcinoma	8204	93	6133	93	1069	95			1002	93
Nonadenocarcinoma	624	7	484	7	62	5			78	7
Gleason grade							<0.001	0.15		
6 or less	355	4	249	4	59	5			47	4
7	1493	17	1050	16	223	20			220	20
8	1578	18	1181	18	200	18			197	18
9	2723	31	2067	31	333	29			323	30
10	606	7	487	7	66	6			53	5
Unknown	2073	23	—	—	—	—			—	—
TNM summary							<0.001	0.22		
M1, any N, any T	6555	74	4841	73	930	82			784	73
M0, N1, any T	1354	15	1078	16	114	10			162	15
M0, N0, T4	919	10	698	11	87	8			134	12
Charlson comorbidity score							0.001	0.17		
None	5153	58	3879	59	646	57			628	58
1	1801	20	1384	21	199	18			218	20
2	863	10	629	10	133	12			101	9
3	460	5	344	5	50	4			66	6
≥4	551	6	381	6	103	9			67	6
Vital status							<0.001	0.13		
Alive	3474	39	2620	40	374	33			480	44
Prostate cancer death	3810	43	2870	43	542	48			398	37
Other causes of death	1544	17	1127	17	215	19			202	19

NHB, non‐Hispanic black; NHW, non‐Hispanic whites.

*P* value calculated from Cochran‐Armitage test for ordinal variables (age group, year of diagnosis, poverty, Gleason grade, CCS) and chi‐square for categorical variables. Percentages calculated from total (including unknown values not displayed).

a Marital status missing for 546 men; poverty indicator missing for 731 men.

## 3 RESULTS

Of the 8828 advanced prostate cancer cases included in this study, 75.0% of men were NHW, 12.8% were NHB, and 12.2% were of some other race/ethnicity. NHB men were significantly younger at the time of diagnosis than NHW men, less likely to be married, and more likely to live in census tracts with 20% or more of residents living at or below the poverty level, and have a greater number of comorbid conditions (all *P* < 0.001). NHB men were slightly less likely than NHW to be diagnosed with Gleason 9 or 10 disease (35% vs 38%, *P* < 0.001), but more likely to be present with distant metastases (M1) at the time of diagnosis (82% vs 73%; *P* < 0.001).

The most common treatment received was ADT (79.5%) either alone or coupled with another treatment (Table 2). Among users, treatment with a gonadotropin‐releasing hormone (GnRH) agonist or antagonist was by far the most common (94.0%) form of ADT with only 6% of men electing to undergo an orchiectomy. Just over 30% of all patients had claims files indicating treatment with radiation and/or chemotherapy, and despite the fact that all patients had evidence of disease spread outside the prostate gland, almost 12% underwent a radical prostatectomy. Denosumab (Xgeva or Prolia) was the most common drug prescribed outside of ADT (13.3%), followed by zoledronic acid (3.2%). Immunotherapy (Sipuleucel‐T) was uncommon (2.7%), as was treatment with radium‐223 (Xofigo; 1.7%), and cryotherapy (0.3%). NHB men were significantly less likely to undergo nearly all forms of treatment compared to NHWs (all *P* < 0.001), with the exception of orchiectomy where they were significantly more likely to undergo the procedure compared with NHW patients (*P* < 0.001). Limiting our analysis to men with Medicare Part D (Pharmacy) coverage did not change the observed racial differences in the prevalence of treatment or use. Among Part D enrollees only, NHW men were more likely to be treated with abiraterone acetate (Zytiga), bicalutamide (Casodex), enzalutamide (Xtandi), and 5α‐reductase inhibitors (Proscar and Avodart) compared with NHBs. Only the progesterone Megace was prescribed more often for NHB men with Part D coverage than their NHW counterparts (24.6% vs 15.0%; *P* < 0.001). Sensitivity analyses performed excluding deaths within 3 months of diagnosis or men without distant metastases also did not alter these patterns (Supplemental Tables S2 and S3).

**Table 2:** Prostate cancer treatment received at any point after diagnosis for NHW and NHB men

	All patients						Part D Coverage				
	NHW		NHB			*P* value	NHW		NHB		*P *value
	N	%	N		%		N	%	N	%	
Total	6617		1131				1988		346		
Radical prostatectomy	779	11.8	80		7.1	<0.001	287	14.4	28	8.1	<0.001
TURP	852	12.9	131		11.6	0.227	231	11.6	40	11.6	0.975
Radiation	2095	31.7	289		25.6	<0.001	636	32.0	84	24.3	0.004
Chemotherapy	1889	28.6	252		22.3	<0.001	547	27.5	68	19.7	0.002
Any ADT	5261	79.5	798		70.6	<0.001	1611	81.0	265	76.6	0.055
Chemical ADT	4944	74.7	706		62.4	<0.001	1527	76.8	236	68.2	<0.001
Orchiectomy	404	6.1	114		10.1	<0.001	105	5.3	35	10.1	<0.001
Other treatments											
Zoledronic acid	213	3.2	23		2.0	0.032	94	4.7	14	4.0	0.577
Sipuleucel‐T	178	2.7	17		1.5	0.019	81	4.1	<11	<2	0.065
Denosumab	880	13.3	114		10.1	0.003	385	19.4	43	12.4	0.002
Radium‐223	112	1.7	<11		<1	0.013	46	2.3	<11	<1	0.036
Cryotherapy	22	0.3	<11		<1	0.909	<11	<1	<11	<1	0.744
No PCa Treatment (does not include oral medication)	693	10.5	209		18.5	<0.001	154	7.7	42	12.1	0.007
Part D drugs											
Bicalutamide							1332	67.0	211	61.0	0.029
Abiraterone acetate							269	13.5	33	9.5	0.041
Enzalutamide							171	8.6	17	4.9	0.020
Megestrol acetate							298	15.0	85	24.6	<0.001
Finasteride							255	12.8	25	7.2	0.003
Dutasteride							132	6.6	14	4.0	0.066
No PCa treatment (including oral medications)							100	5.0	24	6.9	0.145

NHB, non‐Hispanic black; NHW, non‐Hispanic whites; PCa, Prostate Cancer.

Just over 10% of all patients in the study had no evidence of any treatment at any time after diagnosis with NHB men significantly less likely to be treated compared to NHW patients even after adjustment for other covariates associated with the receipt of treatment (aOR = 2.15; 95% CI = 1.70, 2.71; Table 3). Other significant predictors for not receiving treatment included older age, divorced or separated at the time of diagnosis, census tract level % poverty, multiple comorbidities, Gleason score, nonadenocarcinoma histology, and M1 disease (as compared to men with stage any T, N1, M0/MX).

**Table 3:** Predictors for not receiving any prostate cancer treatment^a^ among NHW and NHB men

	Univariate		Multivariate	
	OR	95% CI	OR	95% CI
Age group				
66‐69	ref		ref	
70‐74	1.03	0.83‐1.27	1.04	0.79‐1.37
75‐79	1.25	1.01‐1.55	1.23	0.93‐1.64
80‐84	1.65	1.34‐2.04	1.52	1.13‐2.04
85+	1.98	1.61‐2.44	1.69	1.23‐2.32
Study race				
NHW	ref		ref	
NHB	1.94	1.64‐2.30	2.15	1.70‐2.71
Other	1.22	1.00‐1.49	1.48	1.13‐1.94
Marital status				
Single (never married)	1.56	1.27‐1.91	1.12	0.83‐1.50
Married (including common law)	ref		ref	
Separated or divorced	2.68	2.20‐3.28	2.57	1.97‐3.35
Widowed	1.60	1.33‐1.92	1.07	0.81‐1.40
Poverty indicator (census tract)				
0% to <5% poverty	ref		—	—
5% to <10% poverty	1.04	0.85‐1.27	—	—
10% to <20% poverty	1.19	0.98‐1.45	—	—
20% to 100% poverty	1.66	1.36‐2.02	—	—
Histology			—	—
Adenocarcinoma	ref		—	—
Nonadenocarcinoma	1.89	1.53‐2.34	—	—
Gleason grade				
6 or less	2.41	1.71‐3.39	2.44	1.68‐3.54
7	ref		ref	
8	1.06	0.81‐1.38	1.03	0.77‐1.38
9	1.41	1.12‐1.77	1.39	1.08‐1.79
10	1.43	1.03‐1.98	1.51	1.05‐2.15
AJCC stage summary				
M1, any N, any T	2.17	1.73‐2.73	1.51	1.13‐2.03
M0 or MX, N1, any T	ref		ref	
M0 or MX, N0, T4	1.54	1.13‐2.10	1.23	0.84‐1.82
Charlson comorbidity score				
None	ref		ref	
1	0.66	0.55‐0.80	0.51	0.39‐0.67
2	0.75	0.59‐0.95	0.54	0.38‐0.77
3	1.12	0.85‐1.49	0.80	0.52‐1.23
≥4	1.93	1.54‐2.40	1.06	0.74‐1.52

a No treatment defined as not receiving radical prostatectomy, transurethral resection of the prostate, radiation therapy, chemotherapy, chemical androgen deprivation therapy, orchiectomy, Sipuleucel‐T, denosumab, radium‐223, or cryotherapy.

## 4 DISCUSSION

Findings from this study indicate NHB men initially diagnosed with advanced stage prostate cancer are significantly less likely to undergo any treatment, and the prevalence of use of individual treatments for prostate cancer was consistently lower among these men compared with NHW men with the exception of orchiectomy and use of the progesterone Megace. We observed this despite the fact that all men in the study by virtue of our eligibility criteria were Medicare recipients and thus, presumably, had similar access to treatments. These findings are particularly important given the increase in incidence of distant stage disease among men aged 50 to 69 years, presumably the result of United States Preventive Services Task Force recommendation discouraging routine prostate specific antigen (PSA) screening.^3^, ^13^, ^14^ This investigation is one of the first to report stark differences in treatment uptake by race in a population‐based cohort of men with advanced prostate cancer including the most current treatment modalities for men diagnosed with metastatic disease. Our results are consistent and complement a number of investigations examining treatment disparities among men with both low‐risk prostate cancer and those with high‐risk, but organ‐confined disease.^15^, ^16^, ^17^, ^18^, ^19^, ^20^, ^21^, ^22^

The current standard practice of treatment among men initially diagnosed with stage IV prostate cancer varies by age, the presence of comorbid conditions, whether or not the patient is symptomatic, and with the presence of distant metastases. Most symptoms arise from either the urinary tract or with the presence of bone metastases, and for these men palliative radiotherapy, hormonal therapy, and/or bisphosphonate could be used to manage symptoms.^23^

ADT is prescribed for the majority of men with metastatic prostate cancer at some point during the course of their disease. ADT is effective in slowing the progression of prostate cancer initially, but there are significant side effects associated with ADT including loss of bone mineral density and increased risk of fracture, weight gain, insulin resistance, dyslipidemia, sudden cardiac death, and dementia.^24^, ^25^, ^26^, ^27^ Chemical castration (use of a luteinizing hormone‐releasing hormone (LHRH) agonist [leuprolide or goserelin] with or without other antiandrogens [eg, bicalutamide, flutamide]) is much more common than surgical castration (orchiectomy).^28^ In this study, NHW men were significantly more likely to have received ADT in any form compared with NHB men. However, use of chemical ADT was significantly higher among NHW men (80% vs 71%) while NHB men were significantly more likely to elect to have an orchiectomy (10% vs 6%). Given that orchiectomy results in permanent castration levels of testosterone, one explanation for racial differences in the type of ADT elected could include the potential for more aggressive disease progression in NHB men where orchiectomy might be considered a more effective and complete suppression of testosterone.^29^ It is also possible that NHB men and their physicians elect orchiectomy out of convenience if they have significant barriers, including economic, to regular, frequent visits to receive GnRH injections required as part of long‐term treatment. An analysis of predictors of receipt of orchiectomy included Gleason sum, AJCC stage, marital status, rural residence, census tract poverty level, and age with patterns similar by patient race.

A more effective and recent treatment approach for men diagnosed with metastatic castrate‐sensitive prostate cancer combines ADT with abiraterone acetate, which inhibits testosterone biosynthesis via blocking the action of the CYP17 enzyme. Randomized trials have shown that the addition of abiraterone acetate and prednisone to ADT in metastatic prostate cancer results in improved survival and superior patient‐reported health‐related quality of life (QOL) compared with men on ADT alone.^30^, ^31^ Chemotherapy combined with ADT has been shown to prolong overall survival in men with metastatic disease. In a randomized controlled trial of six cycles of docetaxel 3 weeks apart in addition to ADT, median survival among men with castrate sensitive, metastatic disease randomized to receive docetaxel was 14 months longer than men receiving ADT alone (57.6 vs 44.0 months).^32^ Other predictors of variability in an individual's response to ADT include timing of treatment (immediate vs delayed), use of bisphosphonates, pretreatment PSA levels, testosterone nadir, and time to PSA nadir after treatment. 33, 34, 35, 36

Definitive treatment is employed far less often than hormonal therapy in men with advanced prostate cancer. We observed nearly one‐third of men received radiation therapy and just over 10% underwent radical prostatectomy with significant racial differences in uptake of these treatments. Radiation therapy is sometimes used in men with stage IV M0 disease with at least 6 months of adjuvant ADT,^37^ but also used as part of palliative care to relieve bone pain and improve patient QOL.^38^ A recent meta‐analyses reported that men with metastatic disease who received some local treatment (radical prostatectomy, radiation therapy, and brachytherapy) had significantly better survival at 3 and 5 years compared to men receiving no local treatment.^9^, ^39^ However, because patients treated locally tend to be younger, with more favorable clinical characteristics, it is difficult to attribute the improvement in outcome solely to these treatments.

Once metastatic prostate cancer becomes resistant to hormonal therapy, treatment options become more limited and less effective in slowing the speed of disease progression. Denosumab was prescribed in just over 13% of patients in this study. Denosumab is a human monoclonal antibody that inhibits RANKL, a critical driver of osteoclast formation and survival, thus preventing osteoclast‐mediated bone destruction.^40^, ^41^ Denosumab has been shown to not only reduce skeletal complications in men with castrate‐resistant prostate cancer with bone metastases (mCRPC) with superior efficacy compared with either bisphosphonates or zoledronic acid,^41^, ^42^ but it has also been shown to delay the onset of bone metastases among men with castrate‐resistant, nonmetastatic disease.^43^

Remaining treatments occurred rarely (<5% of patients in this study). Radium‐223 has been shown to be effective in mCRPC by specific targeting and destruction of bone metastases using high‐energy alpha particles. Patients receiving radium‐223 experience lower rates of fracture, bone pain, and spinal cord compression.^44^ Enzalutamide is an androgen receptor inhibitor which has been shown to slow disease progression, delay chemotherapy administration, and improve overall survival in men with mCRPC.^45^ Sipuleucel‐T is an immunotherapy approved by the FDA in 2010 for the management of mCRPC that uses the patient's own cells to stimulate an immune response to prostate acid phosphatase and in turn prostate tumor cells. It has been shown to prolong overall survival in asymptomatic or marginally symptomatic men, absent improvement in other markers of disease progression, and may be used with other therapies.^46^, ^47^, ^48^ Finally, NHB men in our study were significantly more likely than NHW men to have been prescribed the progesterone, megestrol acetate (or Megace). The drug is prescribed to help alleviate hot flashes associated with treatment with ADT or the management of cachexia among men with castrate‐resistant disease.^49^, ^50^

The current study has a number of important strengths. With nearly 9 000 patients, it is one of the largest investigations of treatment patterns in men initially diagnosed with advanced prostate cancer and provides adequate power to detect differences by race in the uptake of treatment even with relatively new, therefore, less prevalent therapies. In addition, the use of claims‐based data reduces the likelihood of treatment misclassification as payment for services rendered requires proper submission of claims. The SEER program is considered the gold standard in cancer surveillance data with significant effort placed on obtaining complete and accurate data on incident cancers. There are some notable limitations. Medicare data can be limited, to some degree, if there is missing information on treatments not covered by or billed to Medicare. It is also difficult in studies which rely upon insurance claims data to tease apart combination therapy or physician intent. Considering the first scenario, for this reason we opted to exclude patients not continuously enrolled in Medicare and those enrolled in a HMO during the study period. In doing so, we limit the generalizability of these findings to non‐HMO patients who tend to be both younger and healthier than Medicare patients.^51^ Remaining possibilities for potential misclassification of treatment include the possibility of one or more treatment paid for completely out of pocket by the patient and those received after conclusion of the study follow‐up period. Analyses to address this show what you might expect in a cohort of men with advanced prostate cancer, that ~98% of patients initiate some treatment within 12 months of diagnosis. So that the potential for misclassification of “no treatment” even among those diagnosed relatively close to the end of study follow‐up would be minimal.

Moreover, despite the fact that all participants in this study are enrolled in Medicare Part A and B, one cannot presume that this translates into equal access to medical care. And, while the trends were similar between all patients in this study and men with Part D benefits that allowed for the investigation of oral prescription medication, we did observe some differences between these two populations. Men with continuous part D coverage were more likely to be younger (*P* = 0.005), unmarried (*P* < 0.001), live in high poverty areas (*P* < 0.001), and have higher Charlson comorbidity scores (*P* < 0.001) compared to men without part D coverage. Furthermore, we were unable to determine if claims for radiation therapy would be considered curative or palliative. One approach suggests that the use of radiation therapy in patients with distant stage disease is all palliative. However, one must also assume that physician intent matches recommended guidelines for use which may not be the case.^52^ Because there were differences by race in survival in this patient cohort, these differences might contribute to the receipt of individual treatments, particularly for men who fail first course treatment (eg, patients who develop castrate‐resistant disease after the use of ADT). Finally, there is some potential for residual confounding in examining predictors for not receiving treatment due to the observational nature of the investigation and the lack of complete information on all confounding factors.

## 5 CONCLUSIONS

NHB men initially diagnosed with advanced prostate cancer were less likely to receive treatment compared with NHW men and, with few exceptions, the use of any individual treatment was lower in NHB men. It is important to extend research into further understanding of predictors of treatment choice, or lack thereof, in this high‐risk population.

## CONFLICT OF INTEREST

The authors declare no conflict of interest.

## AUTHOR CONTRIBUTIONS

Jennifer L. Beebe‐Dimmer: conceptualization, methodology, writing (original draft), and writing (review and editing). Julie J. Ruterbusch: formal analysis, methodology, and writing (review and editing). Kathleen A. Cooney: conceptualization, methodology, and writing (review and editing). Adam Bolton: formal analysis and writing (review and editing). Kendra Schwartz: conceptualization and writing (review and editing). Ann G. Schwartz: conceptualization, methodology, and writing (review and editing). Elisabeth Heath: conceptualization and writing (review and editing).
