# Supplementary material for: Racial differences in patterns of treatment among men diagnosed with de novo advanced prostate cancer: A SEER‐Medicare investigation
Source: Cancer Med. 2019 May 15;8(6):3325–35. doi: 10.1002/cam4.2092 (PMC6558501; doi:10.1002/cam4.2092)
Supplement: Supplementary file 1 [file CAM4-8-3325-s001.docx]

Supplementary Table S1. HCPCs and ICD-9 diagnosis and procedure codes used to identify prostate cancer treatments

| Treatment | HCPCS | ICD-9 Diagnosis Codes | ICD-9 Procedure Codes |
| --- | --- | --- | --- |
| Radical Prostatectomy | 55801, 55810, 55812, 55815, 55821, 55831, 55840, 55842, 55845 |  | 60.3-60.5, 60.69 |
| TURP | 52601 |  | 60.29 |
| Radiation Therapy | G0256, G0261, G0251, G0339, G0340, G0173,  77300-74999, 77520, 77523, 77750-77799 | V58.0, V66.1, V671 | 92.21 – 92.29 |
| Chemotherapy* | Q0083 - Q0085, Q0163-Q0185,  96400-96499, 96500-96529, 96530-96549,  J1675, J3315, J8510, J8520, J8521, J8530-J8999,  J9000-J9999 (excluding codes listed for Chemical ADT) | V581,V662, V672 | 99.25 |
| Chemical ADT | J1675, J1950, J3315, J9202, J9217 - J2919, J9225, J9226 |  |  |
| Orchiectomy | 54520, 54521, 54522, 54530, 54535 |  | 62.4 |
| Zoledronic Acid | Q2051 |  |  |
| Sipuleucel-T | Q2043 |  |  |
| Denosumab | J0897 |  |  |
| Radium-223 | C9399, A9699 |  |  |
| Cryotherapy | G0160, G0161, 55873 |  | 60.62 |

*HCPCS or ICD-9 codes that indicate administration of chemotherapy can be used in conjunction with either chemotherapy or chemical ADT administration; therefore if an administration code claim date was on the same date as a claim for ADT, it would not indicate that chemotherapy was received.

Supplementary Table S2: Prostate cancer treatment received at any point after diagnosis for NHW and NHB men, **excluding men who died within 3 months of diagnosis**

|  | **All Patients** | | | | |  |  | **Part D Coverage** | | | | |
| --- | --- | --- | --- | --- | --- | --- | --- | --- | --- | --- | --- | --- |
|  | **NHW** | | **NHB** | | |  |  | **NHW** | | **NHB** | |  |
|  | **N** | **%** | **N** | **%** | | **p value** |  | **N** | **%** | **N** | **%** | **p value** |
| **Total** | 6,181 |  | 1,048 |  | |  |  | 1,861 |  | 323 |  |  |
|  |  |  |  |  | |  |  |  |  |  |  |  |
| **Radical Prostatectomy** | 779 | 12.6% | 80 | 7.6% | | <.001 |  | 287 | 15.4% | 28 | 8.7% | 0.001 |
| **TURP** | 818 | 13.2% | 127 | 12.1% | | 0.322 |  | 218 | 11.7% | 37 | 11.5% | 0.894 |
| **Radiation** | 2,029 | 32.8% | 271 | 25.9% | | <.001 |  | 617 | 33.2% | 80 | 24.8% | 0.003 |
| **Chemotherapy** | 1,876 | 30.4% | 245 | 23.4% | | <.001 |  | 543 | 29.2% | 67 | 20.7% | 0.002 |
| **Any ADT** | 5,131 | 83.0% | 774 | 73.9% | | <.001 |  | 1,574 | 84.6% | 255 | 79.0% | 0.011 |
| **Chemical ADT** | 4,845 | 78.4% | 691 | 65.9% | | <.001 |  | 1,501 | 80.7% | 227 | 70.3% | <.001 |
| **Orchiectomy** | 373 | 6.0% | 105 | 10.0% | | <.001 |  | 94 | 5.1% | 34 | 10.5% | 0.000 |
| ***Other Treatments*** |  |  |  |  | |  |  |  |  |  |  |  |
| **Zoledronic Acid** | 212 | 3.4% | 23 | 2.2% | | 0.037 |  | 94 | 5.1% | 14 | 4.3% | 0.583 |
| **Sipuleucel-T** | 178 | 2.9% | 17 | 1.6% | | 0.020 |  | 81 | 4.4% | <11 | <5% | 0.065 |
| **Denosumab** | 876 | 14.2% | 114 | 10.9% | | 0.004 |  | 384 | 20.6% | 43 | 13.3% | 0.002 |
| **Radium-223** | 112 | 1.8% | <11 | <1% | | 0.014 |  | 46 | 2.5% | <11 | <1% | 0.036 |
| **Cryotherapy** | 22 | 0.4% | <11 | <1% | | 0.898 |  | <11 | <1% | <11 | <1% | 0.743 |
| **No PCa Treatment**  **(does not include oral medication)** | 461 | 7.5% | 163 | 15.6% | | <.001 |  | 85 | 4.6% | 32 | 9.9% | <.001 |
|  |  |  |  |  | |  |  |  |  |  |  |  |
| ***Part D Drugs*** |  |  |  |  | |  |  |  |  |  |  |  |
| **Bicalutamide** |  |  |  |  | |  |  | 1,300 | 69.9% | 207 | 64.1% | 0.039 |
| **Abiraterone Acetate** |  |  |  |  | |  |  | 269 | 14.5% | 33 | 10.2% | 0.042 |
| **Enzalutamide** |  |  |  |  | |  |  | 171 | 9.2% | 17 | 5.3% | 0.020 |
| **Megesterol Acetate** |  |  |  |  | |  |  | 283 | 15.2% | 83 | 25.7% | <.001 |
| **Finasteride** |  |  |  |  | |  |  | 246 | 13.2% | 25 | 7.7% | 0.006 |
| **Dutasteride** |  |  |  |  | |  |  | 131 | 7.0% | 14 | 4.3% | 0.072 |
| **No PCa Treatment**  **(including oral medications)** |  |  |  | |  | | | 46 | 2.5% | 16 | 5.0% | 0.013 |

Supplementary Table S3: Prostate cancer treatment received at any point after diagnosis for NHW and NHB men, e**xcluding men who were M0 or MX at diagnosis**

|  | **All Patients** | | | | |  |  | **Part D Coverage** | | | | |
| --- | --- | --- | --- | --- | --- | --- | --- | --- | --- | --- | --- | --- |
|  | **NHW** | | **NHB** | | |  |  | **NHW** | | **NHB** | |  |
|  | **N** | **%** | **N** | **%** | | **p value** |  | **N** | **%** | **N** | **%** | **p value** |
| **Total** | 4,841 |  | 930 |  | |  |  | 1,418 |  | 284 |  |  |
|  |  |  |  |  | |  |  |  |  |  |  |  |
| **Radical Prostatectomy** | 35 | 0.7% | <11 | <1% | | 0.922 |  | <11 | <5% | <11 | <5% | 0.535 |
| **TURP** | 647 | 13.4% | 109 | 11.7% | | 0.173 |  | 190 | 13.4% | 35 | 12.3% | 0.625 |
| **Radiation** | 1,465 | 30.3% | 242 | 26.0% | | 0.010 |  | 416 | 29.3% | 72 | 25.4% | 0.175 |
| **Chemotherapy** | 1,514 | 33.1% | 226 | 24.3% | | <.001 |  | 444 | 31.3% | 58 | 20.4% | <.001 |
| **Any ADT** | 3,961 | 81.8% | 666 | 71.6% | | <.001 |  | 1,188 | 83.8% | 222 | 78.2% | 0.022 |
| **Chemical ADT** | 3,672 | 75.9% | 581 | 62.5% | | <.001 |  | 1,110 | 78.3% | 195 | 68.7% | 0.001 |
| **Orchiectomy** | 360 | 7.4% | 106 | 11.4% | | <.001 |  | 96 | 6.8% | 33 | 11.6% | 0.005 |
| ***Other Treatments*** |  |  |  |  | |  |  |  |  |  |  |  |
| **Zoledronic Acid** | 191 | 4.0% | 21 | 2.3% | | 0.012 |  | 87 | 6.1% | 13 | 4.6% | 0.308 |
| **Sipuleucel-T** | 140 | 2.9% | 13 | 1.4% | | 0.009 |  | 65 | 4.6% | <11 | <5% | 0.057 |
| **Denosumab** | 718 | 14.8% | 96 | 10.3% | | <.001 |  | 326 | 23.0% | 40 | 14.1% | 0.001 |
| **Radium-223** | 90 | 1.9% | <11 | <1% | | 0.008 |  | 41 | 2.9% | <11 | <1% | 0.012 |
| **Cryotherapy** | 13 | 0.3% | <11 | <1% | | 0.774 |  | <11 | <1% | <11 | <1% | 0.527 |
| **No PCa Treatment**  **(does not include oral medication)** | 570 | 11.8% | 184 | 19.8% | | <.001 |  | 130 | 9.2% | 37 | 13.0% | 0.046 |
|  |  |  |  |  | |  |  |  |  |  |  |  |
| ***Part D Drugs*** |  |  |  |  | |  |  |  |  |  |  |  |
| **Bicalutamide** |  |  |  |  | |  |  | 1,046 | 73.8% | 187 | 65.9% | 0.006 |
| **Abiraterone Acetate** |  |  |  |  | |  |  | 220 | 15.5% | 30 | 10.6% | 0.031 |
| **Enzalutamide** |  |  |  |  | |  |  | 144 | 10.2% | 15 | 5.3% | 0.010 |
| **Megesterol Acetate** |  |  |  |  | |  |  | 249 | 17.6% | 75 | 26.4% | 0.001 |
| **Finasteride** |  |  |  |  | |  |  | 183 | 12.9% | 20 | 7.0% | 0.005 |
| **Dutasteride** |  |  |  |  | |  |  | 101 | 7.1% | <11 | <5% | 0.025 |
| **No PCa Treatment**  **(including oral medications)** |  |  |  | |  | | | 82 | 5.8% | 20 | 7.0% | 0.414 |
